# Supplementary material for: Acidic enol electrooxidation-coupled hydrogen production with ampere-level current density
Source: Nat Commun. 2023 Jul 14;14:4210. doi: 10.1038/s41467-023-39848-w (PMC10349090; doi:10.1038/s41467-023-39848-w)
Supplement: Supplementary file 1 — Supplementary Information [file 41467_2023_39848_MOESM1_ESM.pdf]

Acidic Enol Electrooxidation Coupled Hydrogen Production with Ampere-level Current Density

Zheng-Jie Chen[1,5], Jiuyi Dong[1,5], Jiajing Wu[2,5], Qiting Shao[1], Na Luo[1], Minwei Xu[1], Yuanmiao Sun[1], Yongbing Tang[1,3], Jing Peng[1,3], Hui-Ming Cheng[1,3,4*]*

[1] Faculty of Materials Science and Energy Engineering / Institute of Technology for Carbon Neutrality, Shenzhen Institute of Advanced Technology, Chinese Academy of Sciences, Shenzhen, 518055, China.

[2] Institute of Information Technology, Shenzhen Institute of Information Technology, Shenzhen, 518172, China.

[3] Shenzhen Key Laboratory of Energy Materials for Carbon Neutrality, Shenzhen Institute of Advanced Technology, Chinese Academy of Sciences, Shenzhen, 518055, China.

[4] Shenyang National Laboratory for Materials Science, Institute of Metal Research, Chinese Academy of Sciences, Shenyang, 110016, China.

[5]These authors were equal major contributors: Zheng-Jie Chen, Jiuyi Dong, Jiajing Wu.

*e-mail: jing.peng@siat.ac.cn; hm.cheng@siat.ac.cn

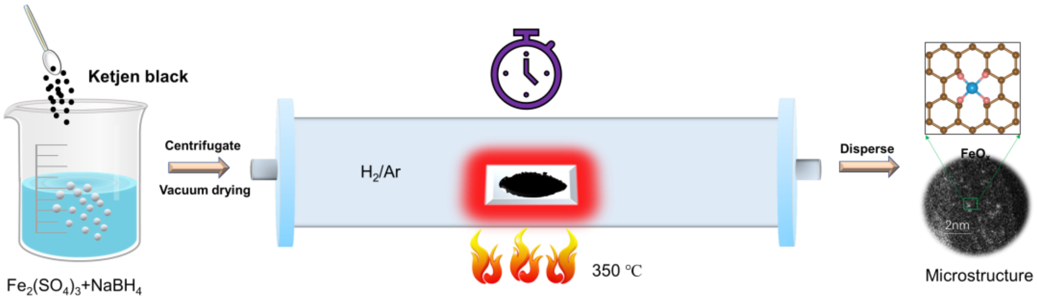

Fig. S1 | Schematic diagram of single-atom and sub-nanocluster preparation.

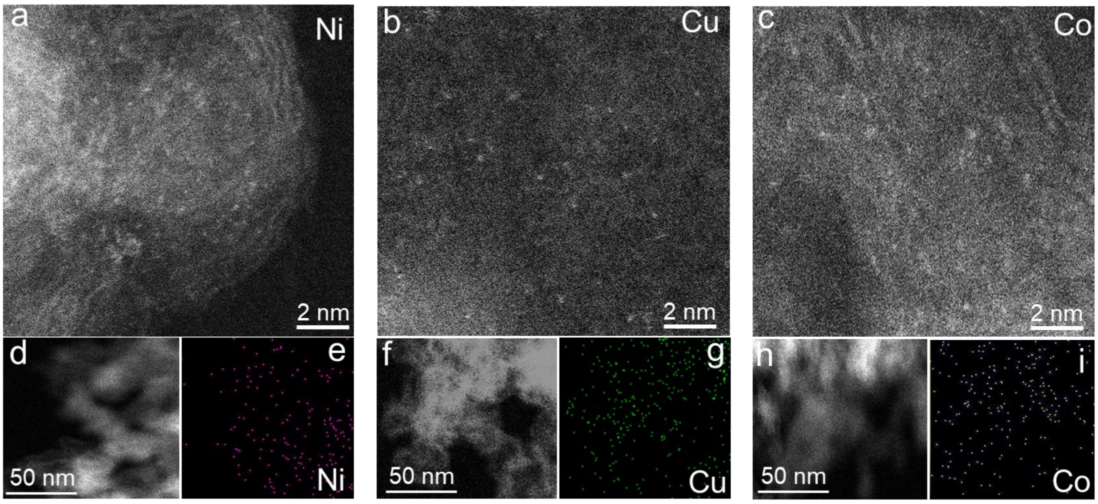

Fig. S2 | AC-HAADF-STEM images for different metal single-atom catalysts. AC-HAADF-STEM images of **a,** Ni **b,** Cu **c,** Co. Corresponding Energy-dispersive X-ray elemental mapping images of Ni **(d-e)**, Cu **(f-g)**, Co **(h-i)**. The dispersions of Ni (purple), Cu (green), Co (yellow). HAADF-STEM and corresponding EDX analysis identified that Ni, Cu, Co are homogeneously dispersed.

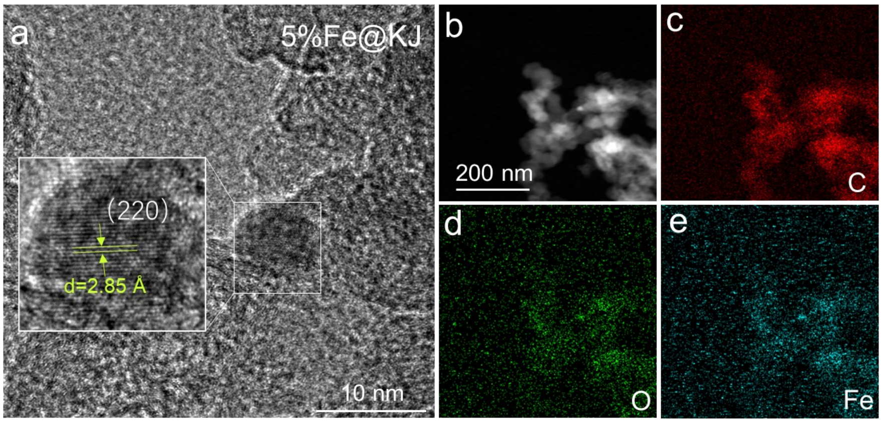

Fig. S3 | Characterizations of 5%Fe@KJ. a, HRTEM images. **b, c, d, e,** HAADF-STEM image and corresponding EDS mapping images of Fe@KJ catalysts. The dispersions of C(red), O(green), Fe(light blue). The elements are very homogenously distributed from the HAADF-STEM and corresponding EDX mapping.

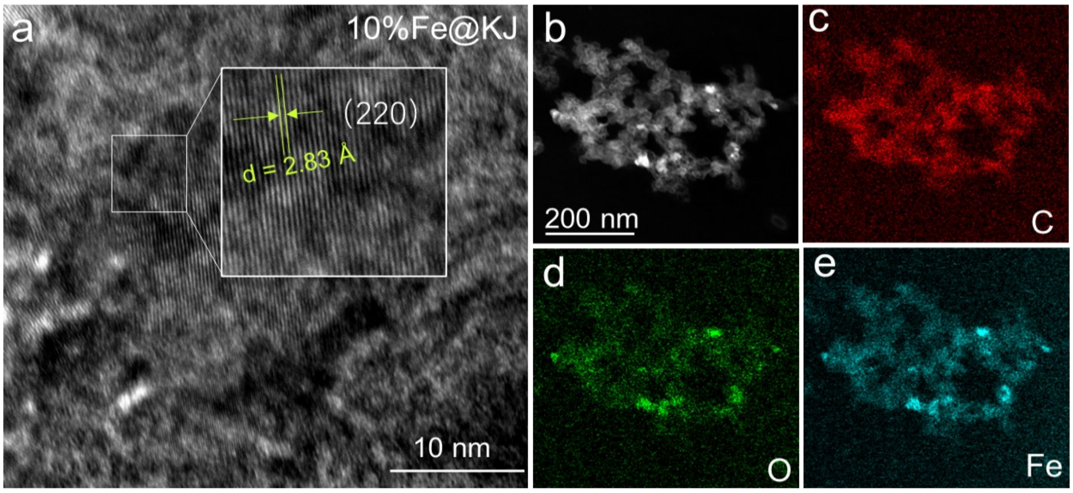

Fig. S4 | Characterizations of 10%Fe@KJ. a, HRTEM images. **b, c, d, e,** HAADF-STEM image and corresponding EDS mapping images of Fe@KJ catalysts. The dispersions of C(red), O(green), Fe(light blue). HAADF-STEM and corresponding EDX with mapping images are homogenously distributed.

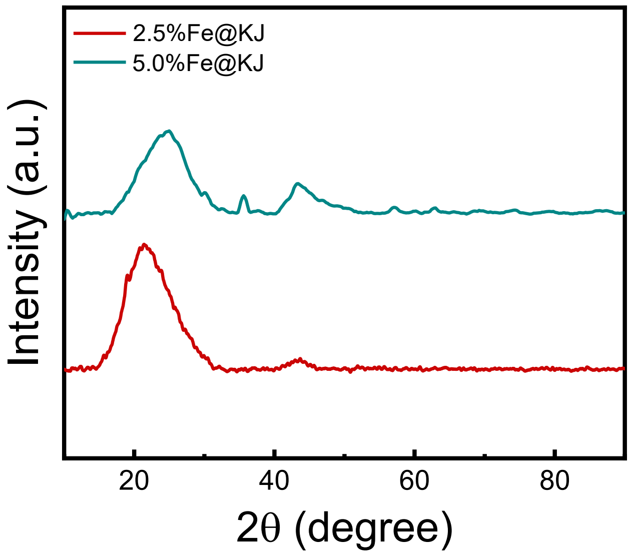

Fig. S5 | XRD for 2.5% and 5%Fe@KJ.

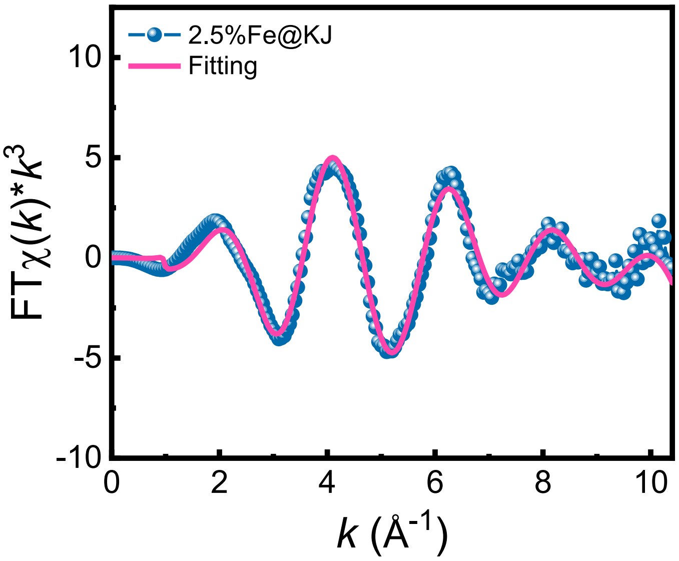

Fig. S6 | Fe K-edge EXAFS (points)and curvefit (line) for 2.5%Fe@KJ, show in k^3 weighted *k*-space.

Supplementary Table 1 | Fe K-edge EXAFS curve fitting parameters

Sample	Path	N	R	$\Delta\sigma^2$	ΔE_0
10%_Fe	Fe-O	**5.1**±0.7	2.01±0.01	0.0114±0.0009	-0.5

N, coordination number; R, distance between absorber and backscatter atoms; σ2, Debye-Waller factor to account for both thermal and structural disorders; ΔE_0, inner potential correction; R factor (%) indicates the goodness of the fit. S_0^2 was fixed to 1.0 as determined from Fe foil fitting. Bold numbers indicate fixed coordination number (N) according to the crystal structure. Fitting range: 2 ≤ k(/Å) ≤ 10 and 1 ≤ R (Å) ≤ 3.0.

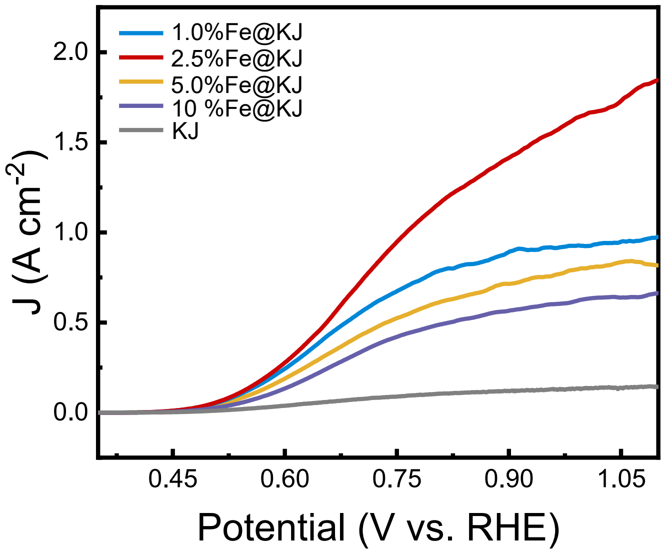

Fig. S7 | LSV with different Fe loadings with 0%, 1%, 2.5%, 5%,10%.

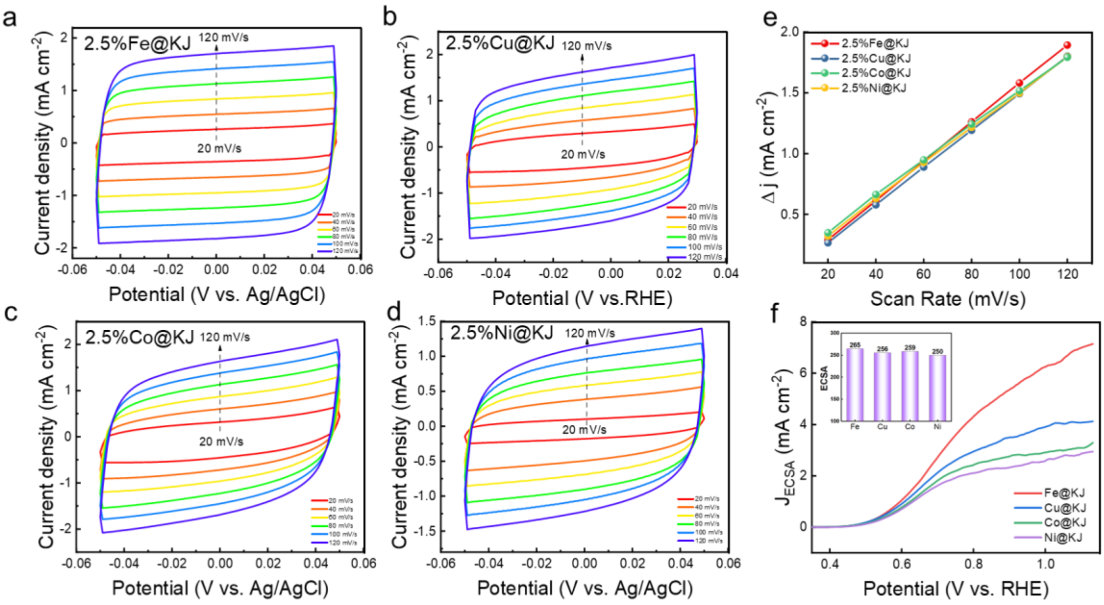

Fig. S8 | C_{dl} for different metal single atoms and the corresponding ECSA. a, b, c, d, CV cycling tests in this voltage range with sweep rates of (20, 40, 60, 80, 100, 120 mV/s). Points are taken at the potentials of 0 V vs. Ag/AgCl. **e,** Scan rate was plotted as a function of the corresponding current density. **f,** The normalized electrocatalytic performance of ECSA. The inset is the ECSA of Fe, Cu, Co, Ni.

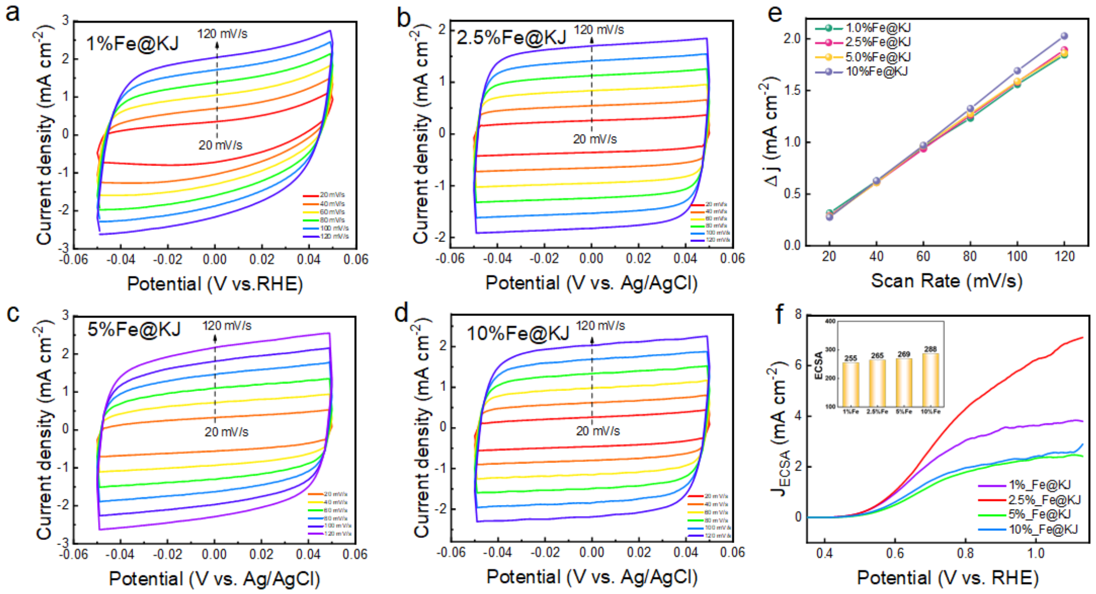

Fig. S9 | C_{dl} for different Fe loadings and the corresponding ECSA. a, b, c, d, CV cycling tests in this voltage range with sweep rates of (20, 40, 60, 80, 100, 120 mV/s). Points are taken at the potentials of 0 V vs. Ag/AgCl. **e,** Scan rate was plotted as a function of the corresponding current density. **f,** The normalized electrocatalytic performance of ECSA. The inset is the ECSA of different loadings of Fe.

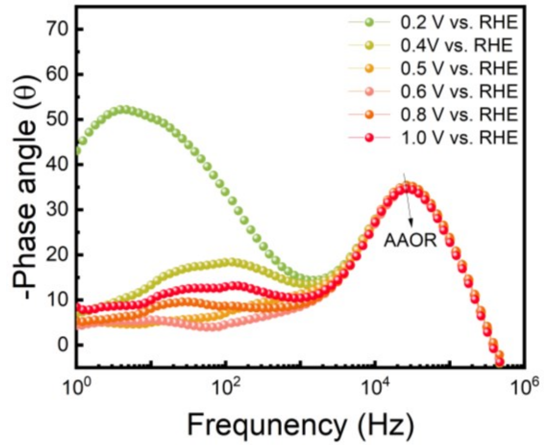

Fig. S10 | Bode plot of different potentials for FeO_δ catalyst.

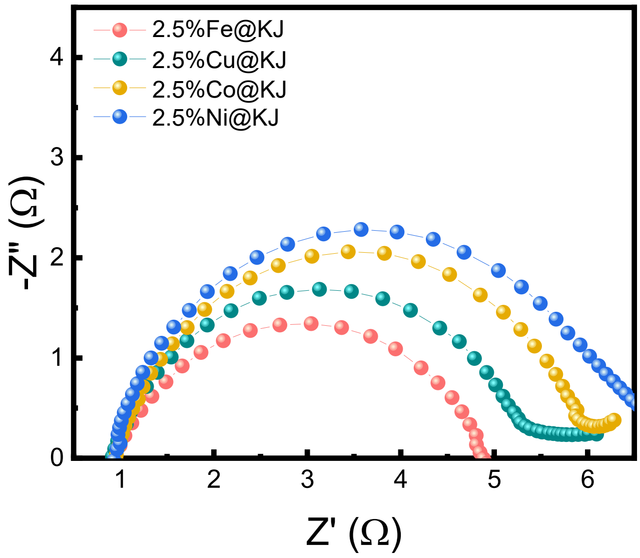

Fig. S11 | EIS of different metals.

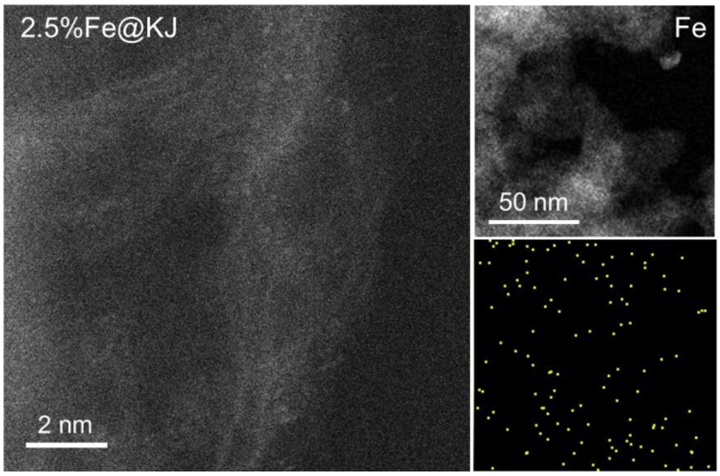

Fig. S12 | AC-HAADF-STEM images for of catalysts after 100 h durability tests.

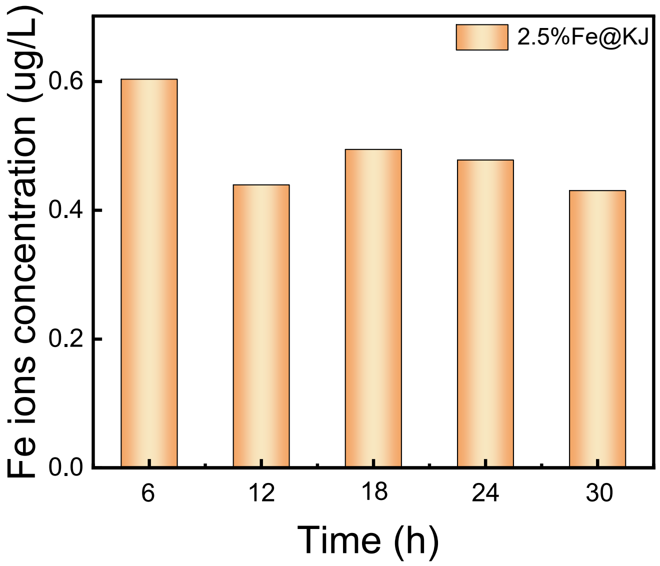

Fig. S13 | Fe ions concentration for different reaction times by ICP-OES.

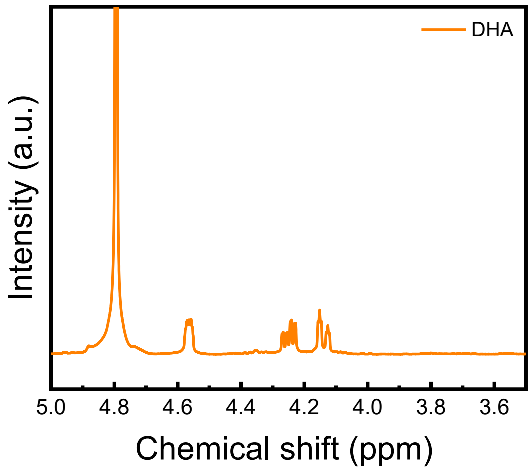

Fig. S14 | ^{1}H NMR spectrum of DHA.

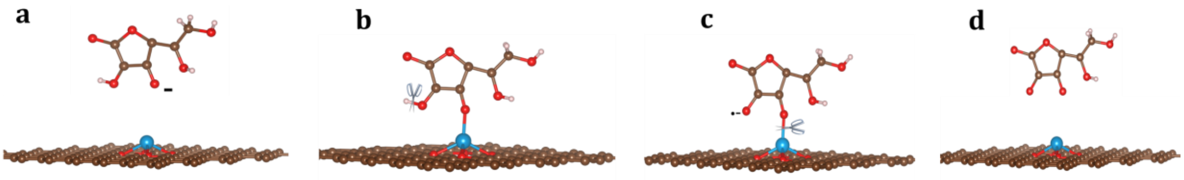

Fig. S15 | The process of DHA production. a, Electrophilic adsorption process of HA^- and M-O-C catalyst. **b,** M-O-C Catalyst adsorbed HA, deprotonation process. **c,** M-O-C Catalyst adsorbed $A^{\bullet -}$. **d,** DHA dissociation.

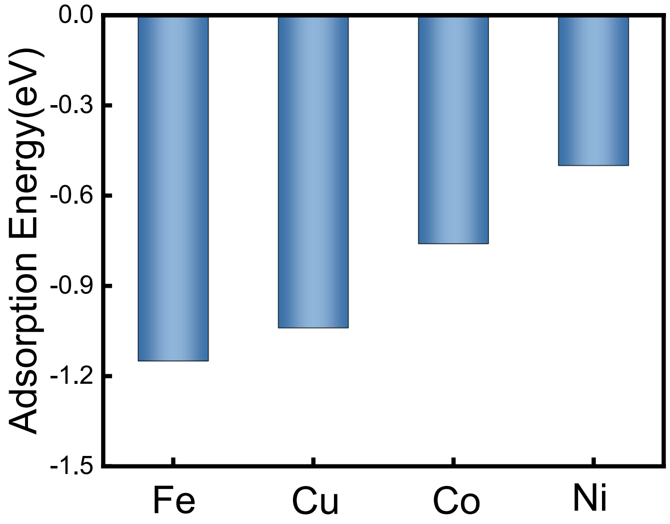

Fig. S16 | Adsorption energy for different metals.

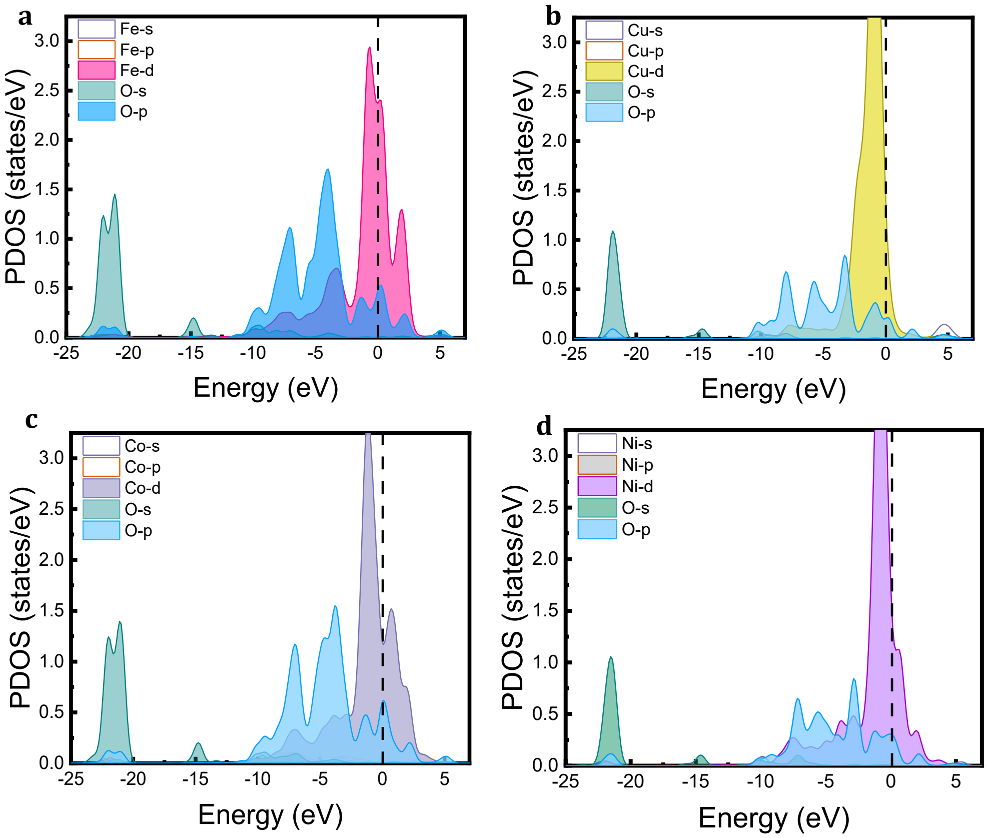

Fig. S17 | Projected density of states for different metals. Pdos of C-O-M (**a,** Fe, **b,** Cu, **c,** Co, **d,** Ni) single atom. Pdos of different catalysts show hybridization orbitals between the 3d orbital of the metal and the O-3p in the enol of HA^-.

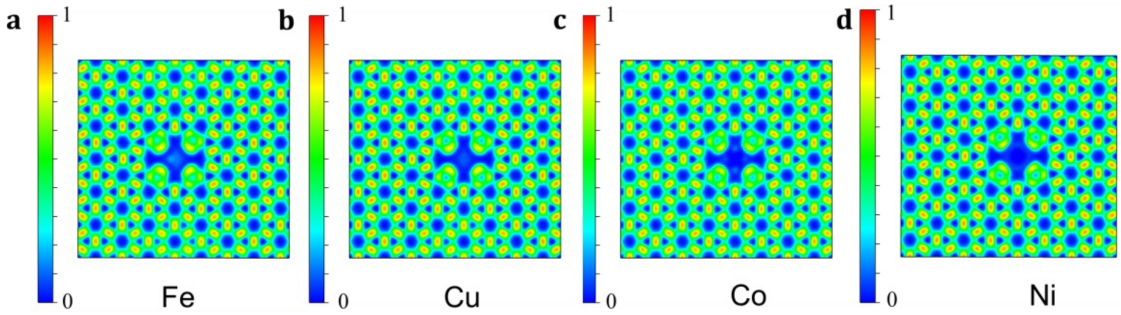

Fig. S18 | Electron localization function for Metal-O-C single atoms. ELF of Fe (**a**), Cu(**b**), Co(**c**), Ni(**d**). The color bar indicates the type of bonding, from blue to red indicating ionic, metallic and covalent bonds between atoms, where ELF less than 0.5 indicates ionic bonding, ELF equal to 0.5 indicates metallic bonding and ELF greater than 0.5 indicates covalent bonding.

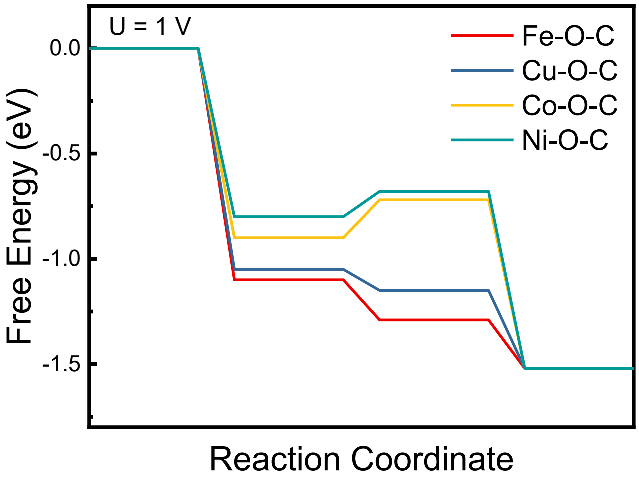

Fig. S19 | Gibbs free energy diagram for different metals at a potential of U = 1 V.

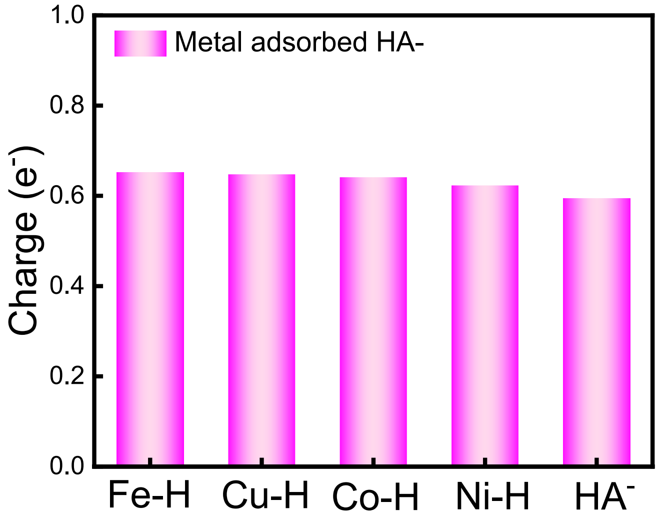

Fig. S20 | Charge transfer of H in the structure of an enol adsorbed with a metal.

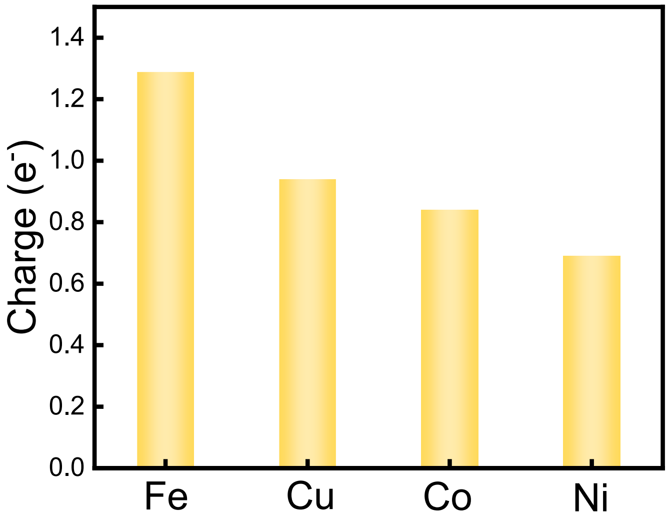

Fig. S21 | Number of charge transfers of different metal single atoms.

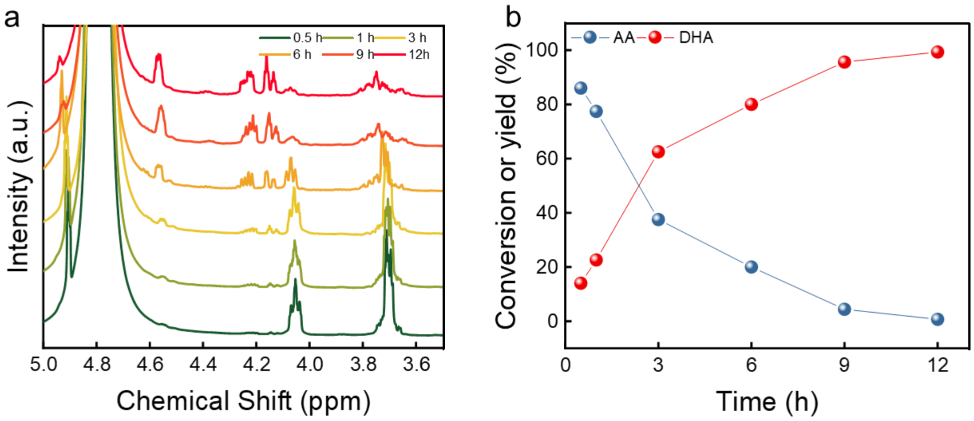

Fig. S22 | ^{1}H NMR characterization and yield of anodic products. a, Production of AA electrooxidation with different operated times in flow cell under a voltage of 0.65 V. **b,** Conversion yield of DHA with different operated times.
