# Supplementary material for: Acidic enol electrooxidation-coupled hydrogen production with ampere-level current density
Source: Nat Commun. 2023 Jul 14;14:4210. doi: 10.1038/s41467-023-39848-w (PMC10349090; doi:10.1038/s41467-023-39848-w)
Supplement: Supplementary file 3 — Reporting Summary [file 41467_2023_39848_MOESM3_ESM.pdf]

nature portfolio

Corresponding author(s): Hui-Ming Cheng

Last updated by author(s): Jun 24, 2023

Reporting Summary

Nature Portfolio wishes to improve the reproducibility of the work that we publish. This form provides structure for consistency and transparency in reporting. For further information on Nature Portfolio policies, see our Editorial Policies and the Editorial Policy Checklist.

Statistics

For all statistical analyses, confirm that the following items are present in the figure legend, table legend, main text, or Methods section.

n/a	Confirmed	
☒	☐	The exact sample size (*n*) for each experimental group/condition, given as a discrete number and unit of measurement
☒	☐	A statement on whether measurements were taken from distinct samples or whether the same sample was measured repeatedly
☒	☐	The statistical test(s) used AND whether they are one- or two-sided<br>*Only common tests should be described solely by name; describe more complex techniques in the Methods section.*
☒	☐	A description of all covariates tested
☒	☐	A description of any assumptions or corrections, such as tests of normality and adjustment for multiple comparisons
☒	☐	A full description of the statistical parameters including central tendency (e.g. means) or other basic estimates (e.g. regression coefficient) AND variation (e.g. standard deviation) or associated estimates of uncertainty (e.g. confidence intervals)
☒	☐	For null hypothesis testing, the test statistic (e.g. *F*, *t*, *r*) with confidence intervals, effect sizes, degrees of freedom and *P* value noted<br>*Give P values as exact values whenever suitable.*
☒	☐	For Bayesian analysis, information on the choice of priors and Markov chain Monte Carlo settings
☒	☐	For hierarchical and complex designs, identification of the appropriate level for tests and full reporting of outcomes
☒	☐	Estimates of effect sizes (e.g. Cohen's *d*, Pearson's *r*), indicating how they were calculated

Our web collection on statistics for biologists contains articles on many of the points above.

Software and code

Policy information about availability of computer code

Data collection	The DFT calculations were carried out by the Vienna ab initio simulation package (VASP)
Data analysis	Origin

For manuscripts utilizing custom algorithms or software that are central to the research but not yet described in published literature, software must be made available to editors and reviewers. We strongly encourage code deposition in a community repository (e.g. GitHub). See the Nature Portfolio guidelines for submitting code & software for further information.

Data

Policy information about availability of data

All manuscripts must include a data availability statement. This statement should provide the following information, where applicable:

- Accession codes, unique identifiers, or web links for publicly available datasets
- A description of any restrictions on data availability
- For clinical datasets or third party data, please ensure that the statement adheres to our policy

The data supporting the results of this study can be found in the article and its supplemental materials. Additional data are available from the corresponding authors upon reasonable request.

Research involving human participants, their data, or biological material

Policy information about studies with human participants or human data. See also policy information about sex, gender (identity/presentation), and sexual orientation and race, ethnicity and racism.

Reporting on sex and gender	There are no sex- and gender-related data in our research.
Reporting on race, ethnicity, or other socially relevant groupings	There are no data reporting on race, ethnicity, or other socially relevant grouping.
Population characteristics	There are no data about the population characteristics of the human research participants in our research.
Recruitment	There are no data involving the human research participants in our research.
Ethics oversight	There are no data involving the human research participants in our research.

Note that full information on the approval of the study protocol must also be provided in the manuscript.

Field-specific reporting

Please select the one below that is the best fit for your research. If you are not sure, read the appropriate sections before making your selection.

☐ Life sciences ☐ Behavioural & social sciences ☒ Ecological, evolutionary & environmental sciences

For a reference copy of the document with all sections, see nature.com/documents/nr-reporting-summary-flat.pdf

Ecological, evolutionary & environmental sciences study design

All studies must disclose on these points even when the disclosure is negative.

Study description	Electrochemical measurement of iron-loaded Ketjen black catalyst (Fe@KJ)
Research sample	iron-loaded Ketjen black catalyst, AA, Na2SO4
Sampling strategy	All electrochemical tests for AA oxidation were performed in 1M Na2SO4 solution on a CHI 660E workstation. The Ag/AgCl and Pt mesh were used as the reference electrode and counter electrode, respectively. Measurements of the three-electrode system were carried out in a membrane-free electrolyzer. The two-electrode system was tested at room temperature and 60? in an electrolyzer, and the Linear voltammetric curves (LSV) curves were obtained in the potential range of -0.2 to 0.8 V vs. AgCl at a sweep rate of 5 mV s-1. The resistance compensation was 80%. All against hydrogen standard electrode potentials (RHE) were from Eq: E(vs. RHE) = E(vs. AgCl) + 0.0591*pH (pH of 1 mol AA is 2.3) + 0.1989. H2 is obtained by the gas collection method of drainage.
Data collection	The solution 1H nuclear magnetic resonance (NMR) was carried out on a 400 MHz NMR spectrometer (Bruker AV400) to to quantitatively identify the oxidation products at different times AA. The product solutions used for the NMR test were obtained at a potential of 0.75 V vs. RHE. The solutions of AA oxidation products of different time measured by IR were extracted at a current density of 10 mA cm-2.
Timing and spatial scale	Jul. 2022-May 2023
Data exclusions	No data were excluded.
Reproducibility	All attempts to repeat the experiment were successful.
Randomization	Not applicable.
Blinding	Not applicable.

Did the study involve field work? ☐ Yes ☒ No

Reporting for specific materials, systems and methods

We require information from authors about some types of materials, experimental systems and methods used in many studies. Here, indicate whether each material, system or method listed is relevant to your study. If you are not sure if a list item applies to your research, read the appropriate section before selecting a response.

Materials & experimental systems

n/a	Involved in the study	
☒	☐	Antibodies
☒	☐	Eukaryotic cell lines
☒	☐	Palaeontology and archaeology
☒	☐	Animals and other organisms
☒	☐	Clinical data
☒	☐	Dual use research of concern
☒	☐	Plants

Methods

n/a	Involved in the study	
☒	☐	ChIP-seq
☒	☐	Flow cytometry
☒	☐	MRI-based neuroimaging
